# The effect of environmental conditions on the occurrence of *Campylobacter jejuni* and *Campylobacter coli* in wastewater and surface waters

**DOI:** 10.1111/jam.15197

**Published:** 2021-07-17

**Authors:** Nicol Strakova, Ekaterina Shagieva, Petra Ovesna, Kristyna Korena, Hana Michova, Katerina Demnerova, Ivana Kolackova, Renata Karpiskova

**Affiliations:** ^1^ Veterinary Research Institute Brno Czech Republic; ^2^ Department of Biochemistry and Microbiology University of Chemistry and Technology Prague Czech Republic; ^3^ Institute of Biostatistics and Analyses Masaryk University Brno Czech Republic

**Keywords:** ammonium salts, *Campylobacter*, chloride salts, seasons, waters

## Abstract

**Aims:**

The purpose of the study was to evaluate the occurrence of *Campylobacter jejuni* and *Campylobacter coli* in the aquatic environment based on the water origin, seasonality and physico‐chemical properties.

**Methods and Results:**

The occurrence of *C. jejuni* and *C. coli* was determined in waste (29) or surface (56) waters in four different seasons. The air and water temperatures were measured during sampling and chemical analyses of water samples for ammonium, chloride, chlorine, nitrite, nitrate, phosphate and iron were performed. The thermotolerant *Campylobacter* spp. were more frequently detected in wastewater (59%; 17 positive samples) compared to surface water (38%; 21 positive samples), with the highest rate in autumn (67% of samples positive) and with a higher *C. coli* occurrence than *C. jejuni* (31% vs. 26%). Ammonium (above 0.2 mg/L) and chloride ion concentrations (above 60 mg/L) favour *C. jejuni*. Similarly, *C. coli* occurrence in water was supported by ammonium (above 0.2 mg/L), chloride (above 60 mg/L) and in addition by phosphate ion concentrations (below 0.7 mg/L).

**Conclusions:**

*Campylobacter* presence in water is influenced by physico‐chemical parameters such as concentrations of ammonium and chloride ions.

**Significance and Impact of the Study:**

Water environment is an alternative source of *Campylobacter*. The concentration of ammonium and chloride ions can be used as a basis for successful prediction of the potential occurrence of *C*. *jejuni* and *C*. *coli* in wastewater and surface water in future.

## INTRODUCTION

Thermophilic *Campylobacter* spp. is the leading cause of gastroenteritis of bacterial origin worldwide (Epps et al., 2013; EFSA, 2019). Unlike other enteric pathogens, e.g., *Salmonella* spp., enteropathogenic *Escherichia coli* and *Shigella* spp. (Nachamkin et al., 2008), *Campylobacter jejuni* and *Campylobacter coli* can cause human gastrointestinal infections with as few as 100 bacterial cells—campylobacteriosis (Tribble et al., 2010). This disease is characterized by nausea, acute diarrhoea, abdominal cramps, fever (Nachamkin et al., 2008), and severe damage of the peripheral nervous system, such as Guillain‐Barré syndrome or reactive arthritis (Koga et al., 2001; Nygård et al., 2004).

Turning to *Campylobacter* spp. in more detail, *C*. *jejuni* and *C*. *coli* are Gram‐negative, microaerophilic, motile, curved or spiral bacteria, but their shape can be changed to coccoid. These coccoid cells represent a degenerative form of *Campylobacter* spp. (https://lpsn.dsmz.de/genus/campylobacter, n.d.).

The environmental conditions, including the properties of water and weather conditions, can influence the presence, survival, shape or even internal factors of *Campylobacter* spp. The most common currently discussed internal factor is biofilm formation (Chmielewski & Frank, 2003; Donlan & Costerton, 2002; Teren et al., 2019). Currently, the effects of external conditions on internal factors were described in *C*. *jejuni* and *C*. *coli* isolates obtained from a wastewater treatment plant. These isolates formed more compact biofilms than isolates from surface waters or isolates from samples of human, food and animal origin (Shagieva et al., 2020).

The main reservoirs of *Campylobacter* spp. are considered poultry and wild birds. *Campylobacter jejuni* and *C*. *coli* are frequently detected as commensal bacteria in broilers, hens (Berndtson et al., 1996) and the prevalence of *C*. *jejuni* in poultry flocks can rise from less than 5% to more than 95% within 7 days (Hartnett et al., 2001). Once poultry are colonized with *Campylobacter* spp., they excrete large numbers of bacteria in their faeces. In this regard, young birds are more susceptible to *Campylobacter* infection. Food‐producing animals, such as pigs and cows, and wildlife such as deer plus other ruminants, may also act as reservoirs of *C*. *jejuni* and *C*. *coli* (Pattis et al., 2017). Campylobacteriosis accounts for an 160 million human cases around the world per year (Kirk et al., 2015). Therefore, *C*. *jejuni* and *C*. *coli* may also be present in other reservoirs.

Currently, there is limited knowledge about *Campylobacter* spp. survival in the aquatic environment. There are several studies that confirmed that water has frequently been identified as *Campylobacter* sources and are often associated with heavy rainfall and intrusion of contaminated waters (Bartholomew et al., 2014; Braeye et al., 2015; Gaardbo Kuhn et al., 2017; Gilpin et al., 2020; Hyllestad et al., 2020; Mouly et al., 2016). What is certain is that *C*. *jejuni* and *C*. *coli* can be found in water springs, rivers, ponds and lakes (Nachamkin et al., 2008). Therefore, human infections might just as well be caused by accidental ingestion of untreated surface water, e.g., during swimming (Sales‐Ortells et al., 2015; Schönberg‐Norio et al., 2004).

Hence, our focus here has been to investigate the occurrence of *C*. *jejuni* and *C*. *coli* in wastewater and surface water, then correlate our findings with environmental conditions, and eventually to determine factors that might influence *Campylobacter* spp. viability in water.

## MATERIALS AND METHODS

### Sample collection

Water sampling was performed according to the EN ISO 19458 standard procedure (Mughini‐Gras et al., 2016). One litre of water was collected and weather conditions (sunny, rainy, etc.) during sampling were recorded. Of 100 samples, 15 duplicates were excluded and the remaining 85 samples were used for analysis, of which 29 samples originated from two different municipal wastewater treatment plants and 56 samples from surface waters, mainly ponds and lakes in 37 different locations of the Czech Republic (Table S1). Wastewater samples (*n* = 29) were collected from the final output of municipal wastewater treatment plants in two locations (A and B) with 16 and 13 samples, respectively (Figure S1). All samples were divided into four groups based on 4 year seasons. Samples were collected in spring (*n* = 30), summer (*n* = 30), autumn (*n* = 18) and winter (*n* = 7). Spring was defined as the period from March 21st till June 20th, summer from June 21st till September 22nd, autumn from September 23rd till December 20th and winter from December 21st till March 20th. Surface water samples were collected from 37 locations (Figure S1). From these, eight locations were selected for repetitive sampling (2–6 times) to cover all seasons. Together with water sampling, the air and water temperatures were measured and the samples were transferred to the laboratory in a cooling box for additional analyses. The temperature of air at the collection sites varied from 2 to 30℃, with the average yearly temperature 15.5℃. Detailed average temperatures of air and water in all seasons are presented in Supplementary data (Table S1). The temperature of all water samples varied from 5 to 30℃ with the average yearly temperature 16.5℃.

### Chemical parameters of water samples

Sample pH values were measured by pH‐Fix PT 4.5‐10 (Macherey Nagel). Chemical analyses of water samples for ammonium, chloride, chlorine, nitrite, nitrate, phosphate and iron were performed semi‐quantitatively by Visocolor Eco kits (Macherey Nagel). The detected pH values of the kit were in the range 4.5–10.0. The concentrations of salts were detected in the following kit intervals: ammonium (NH_4_^+^) 0–3 mg/L, nitrate (NO_3_^−^) 0–120 mg/L, nitrite (NO_2_^−^) 0–0.5 mg/L, chloride (Cl^−^) 0–60 mg/L, chlorine (Cl_2_) 0–0.1 mg/L, phosphate (PO_4_^3−^) 0–5 mg/L and iron (Fe) 0–1 mg/L.

### Detection of *Campylobacter* spp. in poorly filterable water samples by the standard cultivation method

The standard ISO 17995 (Water quality—Detection and enumeration of thermotolerant *Campylobacter* spp.) (International Organization for Standardisation, 2019) was used for the detection of thermotolerant *Campylobacter* spp. with a slight modification (Strakova et al., 2021). Briefly, prefilters with a pore size of 1.4 µm (glass filter; Macherey Nagel) were used to remove mechanical particles from water before sample filtration. Thereafter, water samples (500 ml) were filtered (0.22 µm, mixed cellulose ester filter; Millipore Sigma), and the filters were transferred into two selective broths (Preston and Bolton broth) for enrichment and incubated at 42℃ in an anaerostat (AnaeroJar; Oxoid) under a microaerobic atmosphere (CampyGen 3.5 L; Oxoid). After 44 ± 4 h of incubation, inoculi were cultivated on *Campylobacter* blood‐free selective agar (mCCDA; Modified charcoal‐cefoperazone‐deoxycholate agar) and incubated for another 44 ± 4 h under a microaerobic atmosphere at 42℃, followed by the isolation of presumptive colonies on a nonselective blood agar and species level identification.

### *Campylobacter* species identification

Suspect *Campylobacter* spp. colonies (*n* = 3) on blood agar were identified by multiplex PCR and matrix‐assisted laser desorption/ionisation time of flight mass spectrometry (MALDI‐TOF/MS) (Strakova et al., 2021). Briefly, bacterial DNA was extracted by thermal lysis and PCR was performed using the PPP master mix (Top‐Bio) with primers (Generi Biotech) previously described for the detection of *C*. *jejuni* and *C*. *coli* (Bang et al., 2002; Linton et al., 1997; Winters et al., 1998). MALDI‐TOF/MS (Autoflex speed TOF/TOF; Bruker) was used for confirmation of *C*. *jejuni* and *C*. *coli* identification by spectral comparison with the MALDI Biotyper library (MBT 8468; Bruker).

### Statistical analysis

For statistical analysis, absolute and relative frequencies, median, and percentiles were used to describe categorical and continuous parameters (e.g., place and season sampling, water type, weather conditions, air and water temperature, pH, and 7 ion concentrations), respectively. Crude odds ratios (OR) accompanied by 95% confidence intervals (CI) were obtained by univariate logistic regression models with the aim of assessing the risk of *C*. *jejuni* and *C*. *coli* occurrence in water samples, given different weather conditions (e.g., sunny, rainy, after rain, cloudy) and ion concentrations. Univariate analyses were followed by multivariate logistic regression with backward elimination to obtain adjusted ORs. Decision trees for the best classification and prediction of *C*. *jejuni* and *C*. *coli* occurrence were built using the CHAID growing method. Due to the relatively low number of samples, the data were not divided into training and testing datasets. All data of collected samples were used for creation of trees and crossover validation was applied.

## RESULTS

### The occurrence of thermotolerant *Campylobacter* spp. in the water environment

**FIGURE 1:**
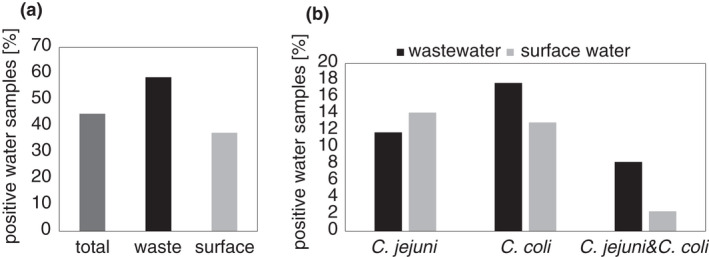
*Campylobacter jejuni* and *Campylobacter coli* isolated from water samples. (a) Percentage of isolated strains from different sample origin. (b) Percentage of isolated *C. jejuni* and *C. coli* strains from wastewater and surface water samples

Of 85 examined samples, 38 (45%) were positive for *Campylobacter* spp. (Figure 1). Briefly, 22 (26%), 26 (31%) and 9 (11%) were *C*. *jejuni*, *C*. *coli* and double positive, respectively (Table 1). Thereafter, *Campylobacter* occurrence in wastewater was further compared to that in surface water, based on seasonality or physico‐chemical water properties (Table S1).

**TABLE 1:** The occurrence of *Campylobacter jejuni* and *Campylobacter coli* in water samples. Number of positive samples and *Campylobacter* isolates collected from waters—total water samples, wastewater samples, and surface water samples. Data (number of collected samples, air temperature, water temperature, and positivity) of surface and wastewater samples collected during four seasons

Samples	No.					%			
	Collected	Total	*Campylobacter jejuni*	*Campylobacter coli*	*C. jejuni and C. coli*	Positive	*C. jejuni*	*C. coli*	*C. jejuni and C. coli*
Total	85	38	22	26	9	44.7	25.9	30.6	10.6
Waste	29	17	10	15	7	58.6	11.8	17.6	8.2
Surface	56	21	12	11	2	37.5	14.1	12.9	2.4

Surface	Collected	*T*_air_	*T*_water_	Positive	
	No.	(°C)	(°C)	No.	%
Season					
Spring	23	20.1	17.8	10	43.5
Summer	22	25.7	22.5	5	22.7
Autumn	8	13.5	11.6	5	62.5
Winter	3	2.7	7.7	1	33.3
All seasons	56			21	**37.5**

Waste	Collected	*T*_air_	*T*_water_	Positive	
	No.	(°C)	(°C)	No.	%
Season					
Spring	7	18.0	18.1	2	28.6
Summer	8	24.8	24.4	6	75.0
Autumn	10	9.4	19.4	7	70.0
Winter	4	7.0	10.0	2	50.0
All seasons	29			17	**58.6**

### The occurrence of *C. jejuni* and *C. coli* in wastewater

Seventeen (58.6%) wastewater samples were positive for *Campylobacter* spp. (Figure 1), and positive detection in samples from both localities was similar, 56% in location A and 62% in location B (Table S1). In detail, 10 (12%) *C*. *jejuni* and 15 (18%) *C*. *coli* strains were isolated from 17 samples. Moreover, 7 (8%) were found double positive for both *Campylobacter* species (Figure 1B—dark column, Table 1—upper part).

### The occurrence of *C. jejuni* and *C. coli* in surface water

In total, 56 surface water samples were collected from 37 locations (Figure S1). to cover all seasons. From these, several locations were selected for repetitive sampling. Samples from 14 locations (38%) were positive for *Campylobacter* spp. Almost 38% of all surface water samples (*n* = 21) were positive for *Campylobacter* spp. (Figure 1). In general, the presence of *Campylobacter* spp. was decreased in surface water when compared to wastewater samples (even 58.6%) (Figure 1a). In detail, 12 (14%) of the surface water samples were positive for *C*. *jejuni* and similarly 11 (13%) were positive for *C*. *coli*. Two samples (2.4%) were positive for both *C*. *jejuni* and *C*. *coli* (Figure 1b—light column, Table 1).

### Seasonality of thermotolerant *Campylobacter* spp. detected in the water environment

**FIGURE 2:**
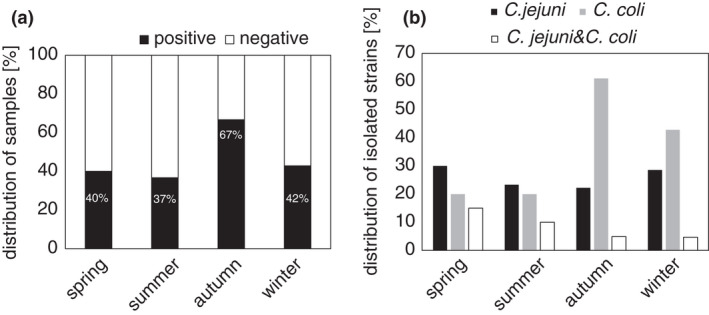
Seasonality of positive detection of *Campylobacter* spp. (a) Distribution of isolated *Campylobacter* spp. per season. (b) Isolated *Campylobacter jejuni* and *Campylobacter coli* strains during four seasons

**FIGURE 3:**
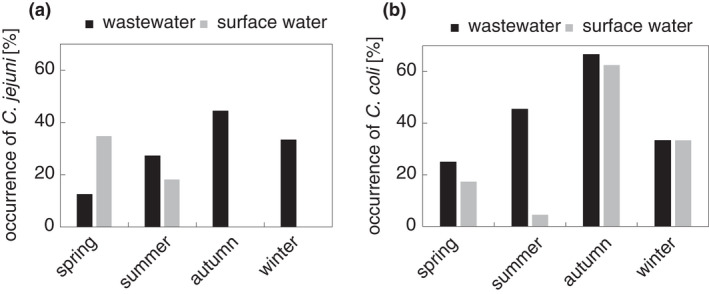
The seasonal occurrence of the particular species of Campylobacters in positive wastewater and surface water samples. (a) Distribution of *Campylobacter jejuni*. (b) Distribution of *Campylobacter coli*. Dark columns—wastewater samples, light columns—surface water sample

In general, the highest air and water temperatures were in summer (Figure S1). *Campylobacter* detection was maximal in surface water in autumn. Two thirds (67%) of autumn samples were *Campylobacter* positive, while in the other seasons only about 40% were positive (Figure 2a). Indiscriminately of water origin our results have shown that the maximal occurrence of *C*. *jejuni* was in spring and winter, while the maximal occurrence of *C*. *coli* was in autumn (Figure 2b). The air and water temperature in autumn had no effect on the presence of *C*. *jejuni* and *C*. *coli* in autumn (Figure S1). Thereafter, we monitored *C*. *jejuni* and *C*. *coli* over four seasons studying wastewater and surface water samples separately (Figure 3). The acquired data were compared by statistical analysis.

### Wastewater seasonality

Wastewater samples were collected during the whole year with a minimum of sample in winter (4) and a maximum in autumn (10). The highest and the lowest temperature of air was reported in summer and in winter, respectively (Figure S1). The temperature of wastewater decreased from summer to autumn, followed by spring and winter. About 75% of summer samples and 70% of autumn‐collected wastewater samples were found *C*. *jejuni* or *C*. *coli* positive. The lowest number of positive samples was detected in spring (28.6%) (Table 1—lower part).

### Surface water seasonality

Unlike wastewater sampling, the number of collected surface water samples differed through the year given that surface water freezes in winter. Surface water samples taken in autumn were most likely to be positive (62.5%), followed by spring (43.5%) and winter (33.3%), while the lowest percentage of positive samples was detected in summer (22.7%) (Table 1).

### Seasonality of the particular species *C. jejuni* and *C. coli*

A general comparison of two *Campylobacter* species occurrence in the wastewater and surface water samples was then made (Figure 3). Both *Campylobacter* spp. were more often present in wastewater (Figure 3; dark column) than in surface water (Figure 3; light column). *Campylobacter coli* was generally more abundant in wastewater than *C*. *jejuni*. *C*. *coli* was also detected in about 60% of samples collected in autumn. In addition, *C*. *jejuni* strains were only isolated from surface water samples in spring and in summer.

### The influence of water properties on the occurrence of *C. jejuni* and *C. coli*

Finally, the occurrence of *C*. *jejuni* and *C*. *coli* in wastewater and surface water was studied in relationship to weather, including two physical parameters (temperature of air and water) and nine environmental chemical properties of all water samples (Table S1). Univariate analyses showed that the risk of *Campylobacter* spp. occurrence was higher in sunny weather with increasing air temperature and with increasing concentrations of ammonium (NH_4_^+^), nitrite (NO_2_^−^) or iron (Table 2). Moreover, multivariate analyses showed that the risk of *Campylobacter* occurrence was increased 4.7 times in wastewater compared to surface water (OR 4.68, 95% CI 1.13–19.41, *p* = 0.034) and that *Campylobacter* risk was higher in water with higher ammonium (NH_4_^+^) concentrations (OR 1.47, 95% CI 1.01–2.15, *p* = 0.044) (Table 2). Enhanced phosphate concentrations (OR 0.2, 95% CI 0.08–0.74, *p* = 0.013) by 1 mg/L decreased the risk of *Campylobacter* spp. occurrence by 76% (Table 2). Other parameters were statistically nonsignificant in the multivariate analysis.

**TABLE 2:** Univariate and multivariate analysis of *Campylobacter* spp. in water samples. Rows: Physico‐chemical properties. Columns: Univariate (crude OR), multivariate analysis (adjusted OR), *p*‐value

	Univariate analysis		Multivariate analysis	
	Crude OR (95% CI)	*p*‐value	Adjusted OR (95% CI)	*p*‐value
Rainy	2.8 (0.26–30.70)	0.102		
Sunny	0.43 (0.15–1.28)	**0.038**		
Air temperature (°C)	0.94 (0.89–0.99)	**0.017**		
Water temperature (°C)	0.94 (0.88–1.01)	0.100		
pH	0.95 (0.40–2.29)	0.910		
NH_4_^+^ (mg/L)	1.42 (1.09–1.84)	**0.007**	1.47 (1.01–2.15)	**0.044**
NO_3_^−^ (mg/L)	1.00 (0.98–1.02)	0.840		
NO_2_^–^ (mg/L)	4.82 (1.01–23.06)	**0.038**		
PO_4_^3−^ (mg/L)	0.86 (0.50–1.46)	0.562	0.24 (0.08–0.74)	**0.013**
Fe^2+/3+^ total (mg/L)	366.8 (0.67–199762.2)	**0.022**	256.0 (0.36–181107.3)	0.098
Cl^–^ (mg/L)	1.02 (0.99–1.05)	0.121		
Cl_2_ free (mg/L)	2515062 (0–3 × 10^16^)	0.447		
Cl_2_ total (mg/L)	0.426 (0.001–210.956)	0.926		

Interestingly, the enhancement of nitrite (NO_2_^−^) by 1 mg/L increased the average risk of *C*. *jejuni* presence 25 times (OR 25.2, 95% CI 1.47–433.3, *p* = 0.026) (Table S2). Otherwise, univariate and multivariate analyses showed that physico‐chemical parameters remained statistically nonsignificant for the risk of *C*. *coli* occurrence (Table S2).

### The prediction of the occurrence of *Campylobacter* spp. in water samples

**FIGURE 4:**
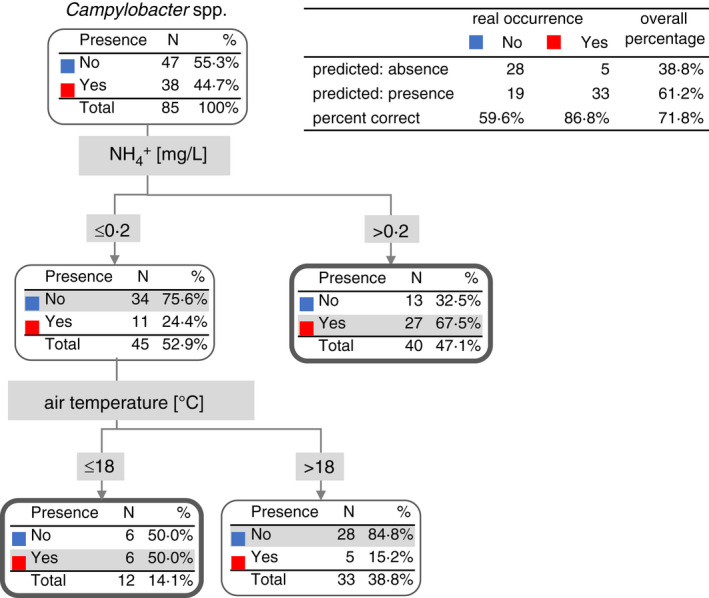
Decision tree for the occurrence of *Campylobacter* spp. based on ion concentrations and wind conditions

Based on the analysis of ion concentrations, we attempted to predict the presence of *Campylobacter* spp. in water. Our results showed that the concentration of ammonium salts increased above 0.2 mg/L supported the occurrence of *Campylobacter* spp. On the other hand, if the concentration of ammonium salts was below 0.2 mg/L, then the air temperature below 18℃ could support the occurrence of *Campylobacter* spp. Based on these two parameters, the total prediction success was 71.8% (Figure 4).

**FIGURE 5:**
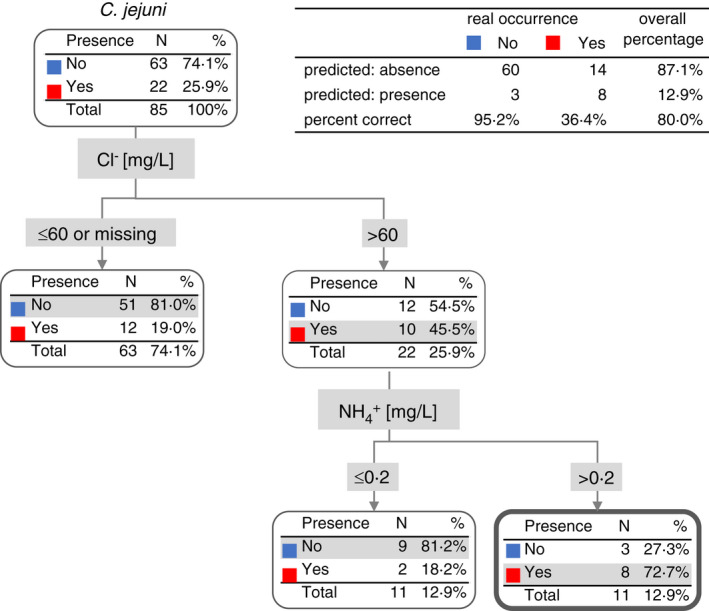
Decision tree for the occurrence of *Campylobacter jejuni* based on ion concentrations

Similarly, the parameters were used for prediction of *C*. *jejuni* (Figure 5). Higher concentrations of chloride over 60 mg/L, together with higher concentrations of ammonium salts over 0.2 mg/L, increased the risk of *C*. *jejuni* occurrence. The total success of prediction of *C*. *jejuni* occurrence in water was 80.0%, based on the presence of chloride and ammonium salts (Figure 5).

**FIGURE 6:**
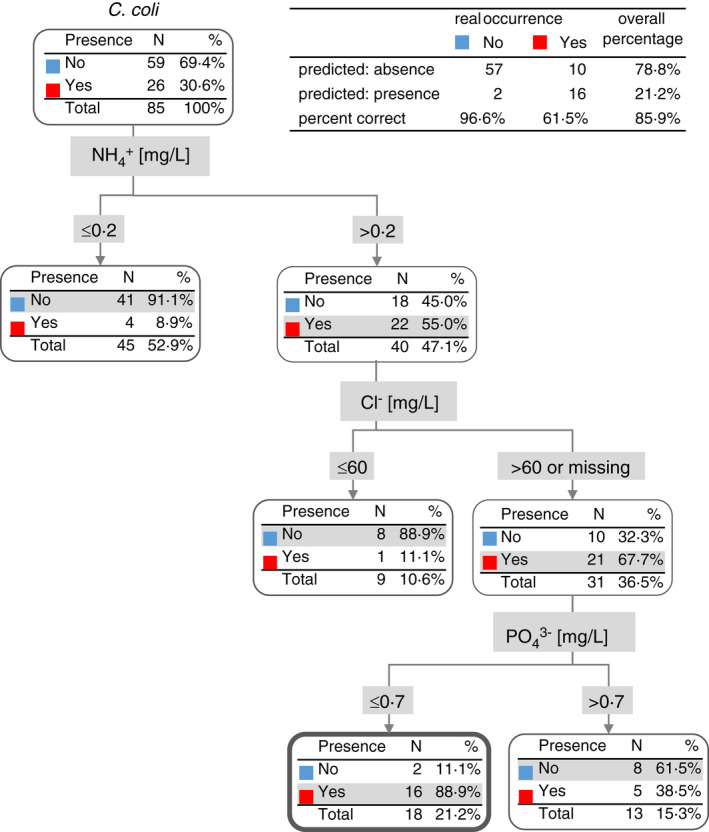
Decision tree for the occurrence of *Campylobacter coli* based on ion concentrations

For the prediction of *C*. *coli* presence (Figure 6) in water, the spectrum of important ions was similar. Lower concentrations of ammonium ions resulted in *C*. *coli* absence. Higher concentrations of chloride over 60 mg/L, together with higher concentrations of ammonium ions over 0.2 mg/L, and lower concentrations of phosphate ions below 0.7 mg/L, increased the risk of *C*. *coli* (Figure 6) occurrence. The total prediction success of *C*. *coli* occurrence was 85.9% based on the chloride, ammonium and phosphate concentration in water samples (Figure 6).

## DISCUSSION

Campylobacteriosis is usually connected to the consumption of undercooked poultry meat or raw milk. The aquatic environment is less well‐described as an alternative source of *C*. *jejuni* and *C*. *coli*. Therefore, our approach here consisted of determining how the occurrence of *C*. *jejuni* and *C*. *coli* varied in both wastewater and surface water samples, according to origin and seasonal variability. The two thermotolerant *Campylobacter* spp. were present in almost 60% of the wastewater samples despite the fact that all tested samples were collected at the final output of municipal wastewater treatment plants. In general, surface water samples were less positive for *C*. *jejuni* and *C*. *coli* than wastewater samples, in accordance with previous findings (Mulder et al., 2020). The strains isolated from wastewater might well originate from the human population because of their excretion from *Campylobacter*‐positive people, especially during late spring, summer and early autumn. On the other hand, surface waters can be contaminated through wild birds or via sewage from farms. When looking at the particular *Campylobacter* species, *C*. *coli* was more abundant than *C*. *jejuni* in water of both origins. These results correlate with the results of a current study from the Netherlands (Mulder et al., 2020). This outcome may be due to its generally higher prevalence in the environment, or due to the higher sensitivity of *C*. *jejuni* to the physico‐chemical characteristics of the environment (Obiri‐Danso et al., 2001).

Several factors, such as the temperature of the water or air, that might contribute to the increased occurrence of *Campylobacter* spp. in water have been considered previously (Cook & Bolster, 2007; Rollins & Colwell, 1986; Thomas et al., 2002). According to our data, we conclude that autumn is the major season for *C*. *jejuni* and *C*. *coli* occurrence in water. Such *Campylobacter* seasonality is also in agreement with the reports of others (Mulder et al., 2020; Nygård et al., 2004). *Campylobacter* spp. can survive for longer periods in water sources during winter, because they are sensitive to UV light and higher temperatures, so bacterial populations may be decimated during the summer (Obiri‐Danso et al., 2001). On the other hand, during the summer period, animals and birds may act as reservoirs for recontamination of water sources, which correlates with the increased incidence of campylobacters between June and November (Nygård et al., 2004). Indeed, autumn but also summer have been shown to be seasons with a higher prevalence of human campylobacteriosis (The European Union One Health 2018 Zoonoses Report, 2019). The enhancement of human campylobacteriosis might also be due to increased consumption of cross‐contaminated food in the summer and autumn during frequent barbecuing, picnics, etc. (Gölz et al., 2014). Therefore, another future study should be performed to compare *Campylobacter* tolerance and sensitivity to temperature, pH, light and oxygen to explain *Campylobacter* water seasonality.

We hypothesized that water composition such as ion concentrations might affect the occurrence of *C*. *jejuni* and *C*. *coli*. They are able to use nitrate (NO_2_^−^) and nitrite (NO_3_^−^) as terminal electron acceptors for growth under severe oxygen‐limited conditions (Pittman et al., 2007). Therefore, *Campylobacter* spp. produce different oxidases and reductases, particularly fumarate reductase, DMSO reductase, nitrite reductase and nitrate reductase, which allow bacteria to grow under low oxygen conditions (Smith et al., 2000). The nitric oxide radical (NO) can arise from nitrogenous species such as nitrite (NO_2_^−^), nitrate (NO_3_^−^) or from ammonium ions (NH_4_^+^) (Pittman et al., 2007), that are the subjects of our study. Ammonium can be later degraded by oxygenation to nitrite (NO_2_^−^) and further to nitrate (NO_3_^−^). A nitrate‐rich diet significantly elevates the exhaled nitric oxide radical (Olin et al., 2001), which is encountered by *Campylobacter* spp. and may influence the outcome of infections. According to univariate analyses of nitrogen‐containing salts, increased levels of ammonium (NH_4_^+^) and nitrite (NO_2_^−^) ions support the presence of *Campylobacter* spp. in water. Moreover, nitrite (NO_2_^−^) increased the risk of *C*. *jejuni* contamination confirming the results of another study where a high concentration of dissolved nitrogen increased the survival of *C*. *jejuni* (Cook & Bolster, 2007). In accordance with the literature, seasonal variabilities in concentrations of nitrite (NO_2_^−^) salts were not observed (Schullehner et al., 2017). However, our multivariate analyses that compared ions mutually supported the proposition that ammonium salts significantly increase the risk of *Campylobacter* contamination in water. The effect of nitrite (NO_2_^−^) was not confirmed in our results, probably due to the overlapping metabolism of ammonium (NH_4_^+^) and nitrite (NO_2_^−^) (Pittman et al., 2007). The increased ammonium salt concentrations above 0.2 mg/L promoted *Campylobacter* occurrence.

It is assumed that iron is an essential element for *Campylobacter* spp. and therefore that iron level could influence *Campylobacter* in water. Surprisingly, the impact of iron concentration on the presence of *C*. *jejuni* and *C*. *coli* in water was not detected either.

Low air temperatures (below 18℃) were also shown to support the occurrence of *Campylobacter* spp. in water when concentrations of ammonium salts were below 0.2 mg/L. Moreover, in the case of *C*. *coli*, lower concentrations of phosphate salts also correlate with the occurrence of these species in water. The total prediction success for *C*. *jejuni* and *C*. *coli* was 80% and 86%, respectively. In view of this fact, further studies will be needed for validation of our data and their wider implementation.

Despite the fact that *Campylobacter* spp. are among the most demanding bacteria requiring specific conditions when cultivated in the laboratory, they are able to survive and multiply in the aquatic environment. We can summarize that the occurrence of *C*. *jejuni* and *C*. *coli* in the aquatic environment was influenced by the origin, seasonality and physico‐chemical properties of waters. In detail, our study showed that wastewaters, ponds and lakes are the reservoirs of *C*. *jejuni* and *C*. *coli*. In addition, 9 (11%) samples were double positive for both species but the occurrence of *C*. *coli* was higher (31%) than *C*. *jejuni* (26%). Despite globally, human campylobacteriosis is caused by thermophilic *C*. *jejuni* (>75%) and, to a lesser extent, *C*. *coli* (Igwaran & Okoh, 2019). It can be explained thus that *C*. *coli* is more commonly associated with waterfowl and environmental sources that have, presumably, been contaminated by them (Sheppard et al., 2009; Sheppard & Maiden, 2015). The year‐round occurrence of *Campylobacter* spp. in water peaking in the autumn demonstrated that *C*. *jejuni* and *C*. *coli* are able to survive temperatures around 8℃ and to aerobic conditions. One health approach suggests that the implementation of efficient regular *Campylobacter* control measures not only in food production but even in water environment, such as in wastewaster treatment plants and surface waters, can help to minimize the occurrence of *Campylobacter* spp. and thereby it can enhance public health safety.

In conclusion, the occurrence of *Campylobacter* in water is influenced by physico‐chemical parameters. Some of these, e.g., the concentration of ammonium and chloride ions, can be used as a basis for successful prediction of the potential occurrence of *C*. *jejuni* and *C*. *coli* in wastewater and surface water in future.

## CONFLICT OF INTEREST

The authors do not have any known competing financial interests or personal relationships that could have appeared to influence the work reported in this paper.

## AUTHOR CONTRIBUTIONS

N. Strakova: Conceptualization, Resources, Investigation, Data curation; Writing—Original Draft Preparation. E. Shagieva: Resources, Investigation. P. Ovesna: Data curation, Resources, Visualization, Writing—Review & Editing. K. Korena: Investigation. H. Michova: Conceptualization, Writing—Review & Editing. K. Demnerova: Conceptualization, Resources. I. Kolackova: Resources. R. Karpiskova: Conceptualization, Resources, Data curation, Writing—Review & Editing, Supervision. All authors contributed to the article and approved the submitted version.

## Supporting information

Figure S1

Table S1

Table S2
