# Supplementary material for: The effect of environmental conditions on the occurrence of Campylobacter jejuni and Campylobacter coli in wastewater and surface waters
Source: J Appl Microbiol. 2021 Jul 17;132(1):725–35. doi: 10.1111/jam.15197 (PMC9290866; doi:10.1111/jam.15197)
Supplement: Supplementary file 1 — Figure S1 [file JAM-132-725-s003.pdf]

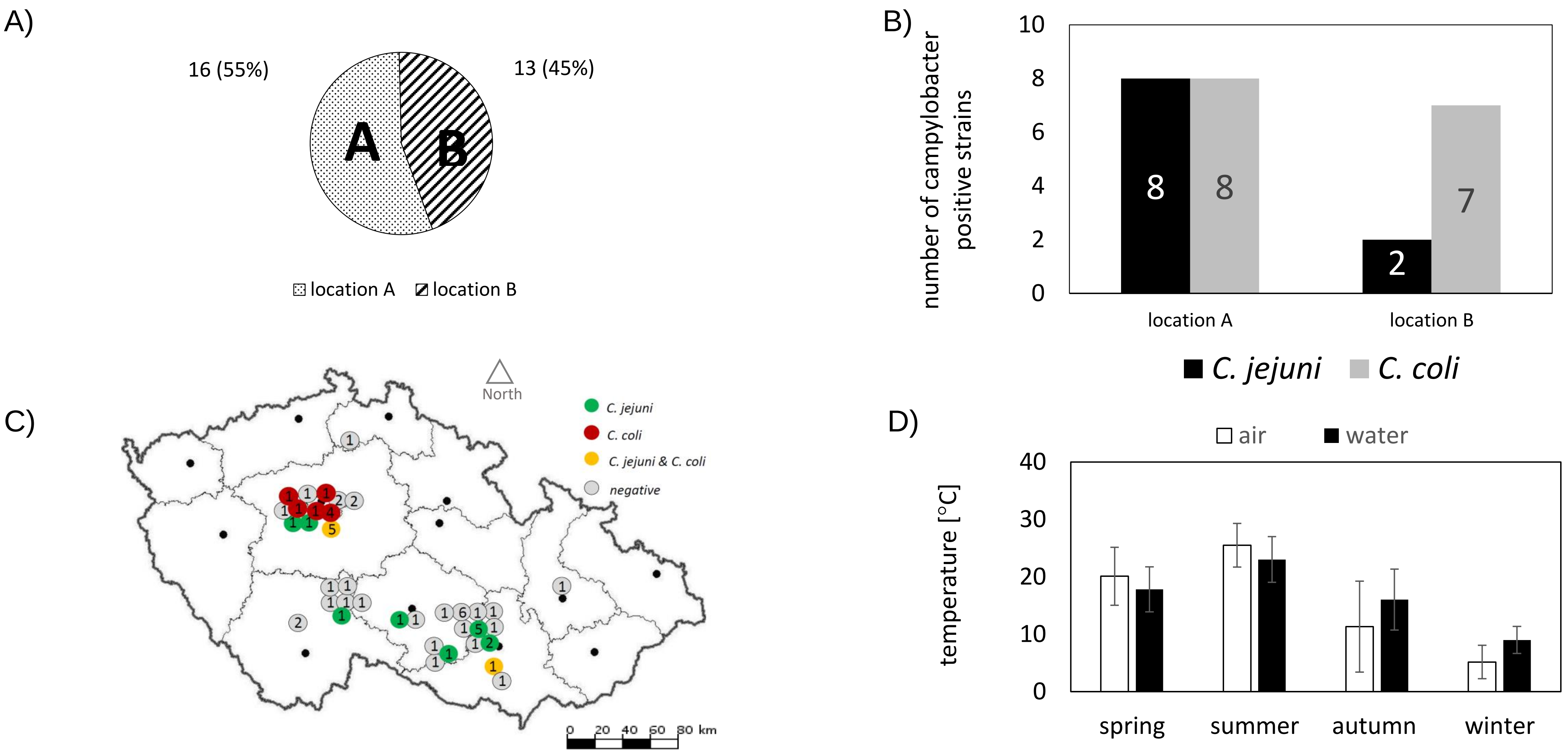

Supplementary Figure 1. Percentage of campylobacter positive water samples. A) Proportion of wastewater samples taken from two municipal wastewater treatment plants - A and B locations. B) Distribution of *C. jejuni* (black) and *C. coli* (gray) strains isolated from wastewater samples according to location. C) A map of water sampling locations with an indication of the number of samples taken per location. The colour indicates the distribution of positive or negative samples per location; Green - *C. jejuni,* red - *C. coli*, yellow - both, gray – negative, black – the regional capitals of the Czech Republic. D) The average air and water temperature [°C] in 4 seasons including SD.
